# The longevity of *Anopheles sundaicus* in a small area: Nongsa Pantai Villages, Batam City, Indonesia

**DOI:** 10.1186/1475-2875-11-S1-P93

**Published:** 2012-10-15

**Authors:** Dewi Susanna, Tris Eryando

**Affiliations:** 1 Department of Environmental Health, Faculty of Public Health, Universitas Indonesia, Kampus Ul Depok, 16424, Indonesia; 2 Department Biostatistic and Health Informatics, Faculty of Public Health, Universitas Indonesia, Kampus Ul Depok, 16424, Indonesia

## Background

Mosquito lifespan is one component of the lifetime transmission potential of an individual mosquito. The length of life or lifespan (longevity) of an adult Anopheles may affect its power of transmitting malaria [1]. This study was to analyze the longevity of *Anopheles sundaicus* in small area, Nongsa Patai Village, Batam City, Indonesia.

## Method

Research used time trend design which investigated within 3-4 months (July-October) in Nongsa Pantai Sub-villages, Nongsa Pantai Village, Batam City, Indonesia. The estimation of *An. sundaicus’s* longevity used a formula based on the Parous Rate and its gonothropic cycle, that is the duration of time the mosquito matures its eggs [2]. The gonothropic cycle of *An. sundaicus* is 3 days.

## Results

The life span (longevity) of *An. sundaicus* in Nongsa Pantai Village ranged from 7.22 to 9.39 with 8.39 in average as shown in Table 1.

**Table 1:** The estimation of longevity of *An. sundaicus* in Nongsa Pantai Village, Batam City, Riau Islands Province during July-October

Observation (month)	Porous rate	Gonothropic cycle (days)	p**	Longevity (days)
July	0.66	3	0.8707	7.22
August*	0.70	3	0.8849	8.41
September	0.71	3	0.8921	8.76
October	0.72	3	0.8962	9.12
*Mean*	0.695	3	0.8857	8.39

*The average of PR in July and September is 0.70%; **p = daily survival rate; equivalen with square root of proportion of female gravid.

Anophelines go through four stages in their life cycle: egg, larva, pupa, and adult The first three stages are aquatic and last 5-14 days, depending on the species and the ambient temperature. The female *Anopheles* is not immediately infective after taking a blood meal and the parasite requires a period of time within the mosquito for its development to an infective stage. The period is termed the extrinsic incubation period [3]. A mosquito needs at least two feedings to complete one transmission cycle [4]. This parameter is strongly dependent on actual air average temperature. It may range from 8 days at 31°C to 22 days at 20°C (the mean value commonly used reaches 15 days). In Berlin, [5] probable extrinsic incubation time 21-24 days calculated for *P. falciparum*[2]. Theoretically, the time required for malaria transmission is at least 13-24 days under control of local temperature and humidity. That can be described as follows: 4-14 day for development from egg to pupae, and the extrinsic incubation period (EIP) ranged 8-10 days assuming the mosquito takes a blood meal directly from an infected person. The longevity of *An. sundaicus* in this area might transmit malaria when the longevity within the 4 month period ranged from 8 days to 10 days and might be the mosquitoes died because of insecticide residual spraying as a vectors control applied.

## Conclusion

The longevity of *An. sundaicus* in coastal area Nongsa Pantai was below the minimum range for the completed life cycle and extrinsic incubation period (EIP).

## References

[B1] Smith DL McKenzie FE Statics and dynamics of malaria infection in Anopheles Malaria Journal 2004 3 13 doi:10.1186/1475-2875-3-13 10.1186/1475-2875-3-13 15180900 PMC449722

[B2] Davidson G Estimation of the survival-rate of anopheline mosquitoes in nature Nature 1954 174 4434 792 793 10.1038/174792a0 13214009

[B3] Zhou SS Huang F Wang JJ Geographical, meteorological and vectorial factors related to malaria re-emergence in Huang-Huai River of central China Malar J 2010 9 337 doi:10.1186/1475-2875-9-337 10.1186/1475-2875-9-337 21092326 PMC3003275

[B4] Kiszewski A Mellinger A Spielman A A global index representing the stability of malaria transmission Am J Trop Med Hyg 2004 70 5 486 498 15155980

[B5] Zoller T Naucke T May J Malaria transmission in non-endemic areas: case report, review of the literature and implications for public health management Malar J 2009 8 71 20 10.1186/1475-2875-8-71 19379496 PMC2672952

